# Attachment Security in Infancy: A Preliminary Study of Prospective Links to Brain Morphometry in Late Childhood

**DOI:** 10.3389/fpsyg.2017.02141

**Published:** 2017-12-12

**Authors:** Élizabel Leblanc, Fanny Dégeilh, Véronique Daneault, Miriam H. Beauchamp, Annie Bernier

**Affiliations:** ^1^Department of Psychology, University of Montreal, Montreal, QC, Canada; ^2^CHU Sainte-Justine Research Center, Montreal, QC, Canada; ^3^Functional Neuroimaging Unit, University of Montreal’s Geriatric Institute, Montreal, QC, Canada; ^4^Center for Advanced Research in Sleep Medicine, Hôpital du Sacré-Cœur de Montréal, Montreal, QC, Canada

**Keywords:** mother–child attachment, infancy, childhood, brain development, social brain, gray matter, volumetrics, cortical thickness

## Abstract

A large body of longitudinal research provides compelling evidence for the critical role of early attachment relationships in children’s social, emotional, and cognitive development. It is expected that parent–child attachment relationships may also impact children’s brain development, however, studies linking normative caregiving experiences and brain structure are scarce. To our knowledge, no study has yet examined the associations between the quality of parent–infant attachment relationships and brain morphology during childhood. The aim of this preliminary study was to investigate the prospective links between mother–infant attachment security and whole-brain gray matter (GM) volume and thickness in late childhood. Attachment security toward the mother was assessed in 33 children when they were 15 months old. These children were then invited to undergo structural magnetic resonance imaging at 10–11 years of age. Results indicated that children more securely attached to their mother in infancy had larger GM volumes in the superior temporal sulcus and gyrus, temporo-parietal junction, and precentral gyrus in late childhood. No associations between attachment security and cortical thickness were found. If replicated, these results would suggest that a secure attachment relationship and its main features (e.g., adequate dyadic emotion regulation, competent exploration) may influence GM volume in brain regions involved in social, cognitive, and emotional functioning through experience-dependent processes.

## Introduction

Seminal work by Harlow and Harlow (1962) suggested that the primate tendency to attach to a caregiver is innate and does not merely reflect physiological needs. Human children attach to a caregiver who is physically present, even if the caregiver does not fulfill a primary physical need, such as feeding, and even if the caregiver adopts abusive behaviors (Bowlby, 1956; Ainsworth, 1967; Cyr et al., 2010). Attachment is a specific, preferential, and enduring emotional tie between an infant and a caregiver, promoting survival and allowing children to feel safe and protected (Bowlby, 1969/1982). Infant attachment is expressed by behaviors such as separation distress, greeting reactions upon reunion, and the tendency to turn to a specific caregiver for reassurance when distressed (Sroufe, 1979; Cassidy, 2016). These innate, universal behavioral tendencies are driven by a biologically based attachment system (Cassidy, 2016). Importantly, however, they are subsequently gradually modulated by caregiver responses, progressively leading to the development of individual differences in the expression and organization of infant attachment behavior (Ainsworth et al., 1978; Fearon and Belsky, 2016). These individual differences are considered to index the “quality” or “security” of attachment relationships. Specifically, a critical tenet of attachment theory is that securely attached children have confident expectations of themselves as being able to solicit the caregiver’s proximity, and of the caregiver as being responsive and available when needed (Bowlby, 1973, 1988). In contrast, infants develop insecure attachments over the course of interactions with caregivers who have difficulty responding adequately to their emotional needs (see meta-analysis by De Wolff and Van IJzendoorn, 1997). Hence, virtually all children become attached to a caregiver, but not all develop secure attachment relationships (Cassidy, 2016).

Decades of longitudinal research have supported the notion that individual differences in infant and child attachment security to primary caregivers are of critical importance for child social, emotional, and cognitive development. Several meta-analyses suggest that variations in attachment security are associated with individual differences in a range of child outcomes: higher attachment security (as compared to insecurity) is associated with better social competence (Groh et al., 2014, 2017a), emotion understanding (Cooke et al., 2016), quality of peer relationships (Pallini et al., 2014), language competence (Van IJzendoorn et al., 1995), as well as fewer internalizing (Groh et al., 2012, 2017a; Madigan et al., 2013) and externalizing behavior problems (Fearon et al., 2010; Groh et al., 2017a).

Such associations between attachment security and child social, emotional, and cognitive development are sometimes interpreted as suggesting that attachment experiences influence the development of children’s brain structures underlying socio-emotional and cognitive functioning (Gunnar et al., 2006; Belsky and de Haan, 2011; Tottenham, 2014). Indeed, although many brain development processes, such as neuronal differentiation, synaptogenesis, and pruning, are guided largely by biological factors (Rakic, 1988), caregiving experiences can also shape brain development in two ways. *Experience-expectant* processes refer to development that occurs in response to experiences that are typically shared by all members of a species (Greenough et al., 1987). Caregiver presence is expected in humans, and indeed caregiver deprivation is associated with alterations of brain structure and function (Eisenberger and Cole, 2012; Tottenham, 2012). Closer to our purposes, *experience-dependent* processes refer to brain development that varies from one person to another as a result of specific individual experiences (Greenough et al., 1987). For example, animal studies indicate that variations in the quality of caregiving have long-term consequences for brain development, notably in brain areas that support stress regulation, social behaviors, and reward processing (Meaney, 2001; Yu et al., 2013; Peña et al., 2014). Accordingly, it is plausible that the security of parent–child attachment, as an important indicator of the quality of the early caregiving environment, may contribute to shaping the developing brain.

This hypothesis is sensible when considering that a core feature of a secure attachment relationship is that the caregiver acts as a secure base from which the child can confidently explore, and seek proximity when distressed (Ainsworth, 1985). During exploration, securely attached children can freely play an active, purposeful role in exploring the surrounding social and physical world, which provides rich stimulation for the developing brain. When, however, they encounter a distressing event during exploration (e.g., hurting oneself while playing), the very nature of their secure attachment relationship allows these children to return to their caregiver for help and soothing (Ainsworth, 1985), which gradually fosters the development of emotion regulation (Calkins, 2004; Cole et al., 2004). Overall, secure attachment relationships are believed to favor both confident exploration and effective emotion regulation in children, which are likely to influence structural development in brain regions involved in a range of social, emotional, and cognitive functions. In fact, it has been proposed that the quality of the attachment bond between children and their caregivers is especially likely to be associated with brain structures underpinning social functioning (Rilling and Young, 2014; Tottenham, 2014), known as the “social brain” and including the superior temporal sulcus, medial prefrontal and anterior cingulate cortices, inferior frontal gyrus, anterior insula, as well as the amygdala (Blakemore, 2008; Adolphs, 2009).

Yet, in contrast to the abundance of research linking attachment security to behavioral outcomes, the links between brain structure and child attachment are still poorly understood (Coan, 2016). Indirect evidence comes from studies of children exposed to maltreatment, which suggest that severely adverse caregiving experiences can lead to morphological alterations in brain regions underpinning social, emotional, and cognitive functions later in life (Teicher and Samson, 2016). Children and adults exposed to childhood maltreatment present abnormal brain volumes and thickness compared to non-exposed individuals in several brain regions (see Lim et al., 2014; Riem et al., 2015, for meta-analyses; Kelly et al., 2013; Whittle et al., 2013; Teicher and Samson, 2016). Nonetheless, these findings should be considered alongside the numerous confounding factors that characterize maltreating families (e.g., poor mental and physical health, poverty, poor quality of sleep, prenatal drug and alcohol use; Edwards et al., 2003; Hussey et al., 2006; Smith et al., 2007; Cuddihy et al., 2013). Given that these factors also influence brain development (Jednoróg et al., 2012; Goodkind et al., 2015; Urrila et al., 2017), the poor quality of parent–child relationships may or may not be the cause of the structural abnormalities observed in the brains of maltreated children (Belsky and de Haan, 2011). Studies in the general population are required to fully understand the association between caregiving experiences and brain morphology.

In contrast to the relatively large body of research on maltreatment, empirical evidence for links between normative variations in parent–child relationship quality and brain development in typically developing children is scarce, and almost all relevant studies have examined parental behavior rather than parent–child relationship quality *per se*. Overall, these studies suggest that normative variations in different dimensions of parental behavior are associated with differences in gray matter (GM) volume and thickness in several brain regions, although directionality varies. Specifically, higher maternal sensitivity has been found to relate to larger subcortical GM volume in infants (Sethna et al., 2017), but also to smaller hippocampal volumes and to (marginally) smaller amygdalar volume in infants (Rifkin-Graboi et al., 2015). Kok et al.’s (2015, 2017) longitudinal studies suggested that parental sensitivity in infancy was not associated with hippocampal and amygdalar volumes in school-aged children, but was associated with larger total GM volume as well as thicker cortex in the bilateral middle frontal gyri, precentral gyri, and left postcentral gyrus. Greater maternal support during the preschool years is associated with larger hippocampal volumes in school-aged children (Luby et al., 2012, 2013, 2016), while self-reported parental praise is related to larger left insula in children aged 5–18 years (Matsudaira et al., 2016). The presence of more positive maternal behavior has been linked to decreased volumetric development in the right amygdala as well as accelerated cortical thinning in the right anterior cingulate and bilateral prefrontal cortices in adolescence (Whittle et al., 2014). On the other hand, negative aspects of parental behavior (e.g., self-reported hostility and observed aggressive behavior) are related to smaller total GM volume (Lévesque et al., 2015) and attenuated cortical thinning in the right superior frontal, superior parietal, and supramarginal gyri, as well as a reduced volumetric development in the left nucleus accumbens in adolescence (Whittle et al., 2016). These brain structures are crucial for children’s social, emotional, and cognitive development, given that they underpin social cognition, emotion regulation, threat detection, attention monitoring, stress regulation and reward processing (Meaney, 2001; Dölen and Malenka, 2014; Frank et al., 2014; Kalisch and Gerlicher, 2014; Deen et al., 2015).

In light of the growing literature pertaining to specific dimensions of parental behavior and brain morphology, it is surprising that almost no research has focused directly on the quality of the parent–child dyadic relationship, of which attachment security is perhaps the best documented and most widely recognized indicator. Given that the quality of parenting behavior is moderately associated with parent–child attachment security (De Wolff and Van IJzendoorn, 1997), the body of literature presented above suggests that parent–child attachment security may relate to children’s brain morphology. Yet, to our knowledge, only two studies have examined the relations between brain structure and the quality of parent–child attachment relationships, and both focused on subcortical volumes (amygdala, hippocampus, caudate nucleus, thalamus) once participants reached adulthood. These studies suggest that poorer attachment quality to mother in infancy (assessed with the Strange Situation Procedure; SSP, Ainsworth et al., 1978) relates to larger volume of the amygdalae in adulthood (Moutsiana et al., 2015; Lyons-Ruth et al., 2016). These two longitudinal studies highlight the potentially long-lasting link between early parent–child attachment and subcortical brain structure. However, it is not known whether the longitudinal links are already apparent during childhood, whether the direction of association is stable, and whether attachment may also relate to other brain regions. These are important questions in light of increasing evidence that developmental considerations play a crucial role in the links between caregiving experiences and regional brain development, including directionality of such links (Tottenham and Sheridan, 2009; Teicher et al., 2016). For example, higher-quality parenting is associated with smaller hippocampal volumes in infants and children [Luby et al., 2012, 2013, 2016; Rifkin-Graboi et al., 2015; but see Sethna et al.’s (2017) results indicating larger subcortical GM volume]; however, higher-quality parenting is associated with larger hippocampal volume in adolescence (albeit in a sample of children exposed to cocaine during gestation; Rao et al., 2010).

Building on previous studies (Moutsiana et al., 2015; Lyons-Ruth et al., 2016), the current report examines the longitudinal associations between mother–infant attachment security and whole-brain GM volume and thickness in late childhood. Previous studies have used an *a priori* regions-of-interests approach to investigate the links between parent–child relationship quality and brain structure, which may limit the scope of the conclusions that can be drawn; a whole-brain approach was therefore used here. We assessed early mother–child attachment security with the Attachment Behavior Q-Sort (AQS; Waters and Deane, 1985), which yields a continuous score for attachment security rather than assignment to a particular attachment category. This approach maximizes statistical power by affording excellent detection of fine individual differences, and may be especially appropriate in the context of small sample sizes (Groh et al., 2017b). Psychometric work also suggests that a dimensional approach is coherent with the underlying structure of individual differences in attachment (Fraley and Spieker, 2003). Given the scarcity of literature on attachment security and brain morphology in typically developing children, and the fact that a large number of brain regions have been variously linked to caregiving experiences, the statistical analyses were exploratory and no *a priori* hypotheses were formulated with regards to the location of putative associations or the direction of associations, considering also that some aspects of brain development trajectories follow an inverted U-shape (Shaw et al., 2008; Giedd et al., 2015).

## Materials and Methods

### Participants

Participants included in the present study (*n* = 33) were followed annually as part of a larger longitudinal research project that documents the prospective associations between the early caregiving environment and several facets of child development (see Bélanger et al., 2015). In the present study, we report on attachment security assessed at 15 months of age (T1; *M* = 15.65, *SD* = 0.97, range = 14.50 – 18.00) and structural magnetic resonance imaging (MRI) data collected when children were 10–11 years of age (T2, *M* = 10.59, *SD* = 0.46, range = 10.0 – 11.67 years). The study was approved by the local research ethics committee of aging-neuroimaging of the CIUSSS du Centre-Sud-de-l’île-de-Montréal and all families provided written informed consent for participation.

Families were recruited from random birth lists of a large Canadian metropolitan area, provided by the Ministry of Health and Social Services. Inclusion criteria for participation were full-term pregnancy (i.e., at least 37 weeks of gestation) and the absence of any known physical or mental disability, severe developmental delay in the infant, acquired brain injury, and standard MRI counter-indications. For the current analyses, 64 families were invited to participate in structural MRI when children reached 10 years of age; among them, 35 (54.69%) agreed to participate. Families who agreed to participate (*n* = 35) did not differ from those who refused (*n* = 29) in terms of family income, parental age, education, and ethnicity, as well as child attachment security to mother in infancy (all *p*s > 0.21, see **Table 1**). Of the 35 families who agreed to take part in the MRI protocol, one child was excluded from the analyses because of excessive head motion (translation > 2.5 mm or rotation > 2.5 degrees) and one because of suspected neuropathology. Consequently, data from 33 children [20 girls and 13 boys; χ^2^(1) = 1.46, *p* = 0.23] were used in the analyses. Group comparisons between families included in the analyses (*n* = 33) and those who declined the MRI protocol (*n* = 29) were not significant.

**Table 1:** Sociodemographic information and attachment security scores for families who accepted vs. declined participation in the magnetic resonance imaging (MRI) protocol.

	Accepted MRI *n* = 35	Declined MRI *n* = 29	Group comparisons
**Parental age at recruitment**			
Mothers	31.63 ± 5.05	32.02 ± 3.50	*t*(62) = -0.36; *p* = 0.73
Fathers	33.40 ± 5.29	34.07 ± 4.86	*t*(62) = -0.52; *p* = 0.60
**Parental years of education**			
Mothers	15.40 ± 2.23	15.26 ± 2.32	*t*(62) = 0.24; *p* = 0.81
Fathers	15.60 ± 1.94	14.97 ± 2.10	*t*(62) = 1.30; *p* = 0.21
**Ethnicity**			
Mothers	80.00	86.21	χ^2^(1) = 0.43; *p* = 0.51
Fathers	74.30	75.90	χ^2^(1) = 0.02; *p* = 0.89
Family income	74.29	79.31	χ^2^(1) = 0.22; *p* = 0.64
Language at home	80.00	82.76	χ^2^(1) = 0.08; *p* = 0.78
Attachment security	0.48 ± 0.26	0.50 ± 0.20	*t*(62) = -0.42; *p* = 0.67

*For ethnicity, family income, and language, values represent percentages of families with a Caucasian mother/father, an income above $60,000, and French as the main language. For parental age, parental education, and attachment security, values represent mean ± standard deviation. Two children who underwent MRI were excluded from main statistical analyses (*n* = 33): excluding them from the group comparisons did not change the results.*

### Attachment Security Assessment

Mother–infant attachment security was assessed at T1 using the Attachment Behavior Q-Sort (AQS; Waters and Deane, 1985). The observer-version of the AQS is considered one of the gold-standard measures of attachment (Van IJzendoorn et al., 2004) as it shows excellent construct validity, converging with attachment security assessed with the SSP, with child socio-emotional adaptation, and with maternal sensitivity (see Van IJzendoorn et al., 2004; Cadman et al., 2017 for meta-analytic evidence), while also demonstrating discriminant validity with respect to child temperament (Cadman et al., 2017). In fact, meta-analytic data suggest that the AQS is more closely related to child outcomes than the SSP (Fearon et al., 2010), which makes it an instrument of choice to study putative associations between early attachment and brain morphology.

In this study, trained research assistants observed infant behaviors throughout a 70- to 90-min home visit modeled after the work of Pederson and Moran (1995). This visit was purposely designed to create a situation during which maternal attention was solicited by both infant demands and research-related tasks (e.g., mothers had to fill in questionnaires while infants were not cared for by the research assistant). This aimed at challenging mothers’ capacity to divide their attention between competing demands, thus reproducing the natural conditions of daily life when caring for an infant. Restricting maternal availability to infant demands is a classic trigger of the attachment system in infancy (Ainsworth et al., 1978). The research assistants completed the AQS immediately after the visit. In order to maximize the reliability of the observations performed during these home visits, which was central to this study, we followed Pederson and Moran’s (1995) recommendations for training our home visitors. Research assistants first attended a 2-day training workshop on techniques of home visiting and structured observation of mother–infant interactions. They reviewed several videotapes to practice coding the AQS. The assistants then performed their first few home visits with a more experienced colleague, and the two completed the AQS together. When the junior home visitors were deemed ready to lead home visits independently, the next two or three visits were followed by a debriefing session with an experienced graduate student, to review the salient elements of the visit before scoring the AQS. Inter-rater reliability testing (described below) took place only after assistants had successfully completed this training.

The AQS consists of 90 items measuring the quality of the child’s attachment behaviors toward a specific figure (the mother in this case). Each item of the AQS describes a potential child behavior. Based on observations performed during the entire home visit, research assistants sorted those behaviors into nine clusters of 10 items each, ranging from “very similar” to “very unlike” the observed child’s behaviors. The global score for attachment security consists of the correlation between the observer’s sort of the 90 items and a criterion sort for the prototypically secure infant (Waters and Deane, 1985). Attachment security scores can thus range from -1.0 (highly insecure) to 1.0 (highly secure). Prototypical security represents a fluid balance between exploration of the environment and appropriate reliance on the caregiver for support when needed. To examine inter-rater reliability, 23.1% of the home visits were conducted by two research assistants, who then completed the AQS independently. Agreement between the two raters’ sorts was satisfactory, intra-class correlation (ICC) = 0.71.

### Pubertal Status

A parent-report version of the rating scale for pubertal development (Carskadon and Acebo, 1993) was completed at the time of the MRI (T2). Parents evaluated their child’s pubertal development using a scale ranging from 1 = “not yet started” to 4 = “seems completed.” Children’s pubertal status was derived from three items for both boys (body hair growth, voice change, facial hair growth) and girls (body hair growth, breast development, menarche), as described by Carskadon and Acebo (1993).

### Structural Magnetic Resonance Imaging

#### Acquisition

Neuroimaging data were collected at T2 using a 32-channel head coil on a Siemens 3 Tesla scanner (MAGNETOM Trio, Siemens, Erlangen, Germany). Structural data were acquired using a three-dimensional T1-weighted 4-echo magnetization-prepared rapid gradient-echo sequence [3D-T1-4echo-MPRAGE sagittal; repetition time (TR): 2530 ms; first echo time (TE): 1.64 ms; echo spacing ΔTE: 1.86 ms; flip angle: 7°; 176 slices; slice thickness: 1 mm; no gap; matrix: 256 × 256; field of view (FoV): 256 mm; in-plane resolution: 1 mm× 1 mm; duration: 363 s].

#### Pre-processing

Pre-processing for the voxel-based morphometry (VBM) and the surface-based morphometry (SBM) analyses were performed using the SPM12 package (Statistical Parametric Mapping, Institute of Neurology, London, United Kingdom) and the CAT12 Toolbox^1^ running on MATLAB version R2016a (MathWorks, Inc., Natick, MA, United States). For VBM, T1-weighted images were segmented into GM, white matter (WM), and cerebrospinal fluid (CSF) using age-appropriate stereotaxic tissue probability maps (NIHPD 7.5-13.5 asymmetric^2^; Fonov et al., 2011). Pediatric templates were used to minimize the potential confounds introduced by developmental differences in cortical morphometry (Yoon et al., 2009). Next, the segments were spatially normalized to the Montreal Neurological Institute (MNI) space with a voxel size of 1.5 mm × 1.5 mm × 1.5 mm. Finally, the resulting GM maps were modulated and smoothed with 8-mm full-width-at-half-maximum (FWHM) smoothing kernels. For SBM, T1-weighted images were segmented and spatially normalized as for VBM. The cortical surface was reconstructed from volumetric data using the projection-based thickness method. The cortical thickness maps were resampled onto the cortical surface and smoothed with a standard 15-mm FWHM smoothing kernel.

#### Statistical Analyses

The threshold-free cluster enhancement (TFCE) method implemented in CAT12 was used to identify statistically significant clusters. TFCE is a cluster-based thresholding method that overcomes the problem of choosing an arbitrary cluster-forming threshold, while keeping the sensitivity advantage of cluster-based thresholding (Smith and Nichols, 2009). TFCE uses a permutation approach that maximizes statistical power in small sample studies (Pernet et al., 2015). Using 5,000 permutations and non-parametric testing, a voxel-wise *p*-value map is produced. An explicit GM mask based on the mean normalized GM images of all participants was used to ensure that the analyses were restricted to GM. Resulting statistical maps were thresholded at *p* < 0.05 corrected for multiple comparisons by false discovery rate (FDR; Chumbley et al., 2010).

### Main Analyses

The main analyses focused on GM volume and thickness. A multiple regression analysis was performed using CAT12 to predict GM volumes in late childhood from attachment security in infancy, after accounting for confounding variables (described below). Similar analyses were performed to predict cortical thickness, and right and left hemispheres were analyzed separately. In order to account for differences in overall brain size, total intracranial volume (ICV) was controlled for in the VBM analyses (Barnes et al., 2010; Malone et al., 2015). As ICV is not related to cortical thickness (Toro et al., 2008; Winkler et al., 2010), it was not controlled for in the SBM analyses. Child age and sex, pubertal status, as well as maternal education are associated with cortical volume and thickness (Barnes et al., 2010; Blakemore et al., 2010; Jednoróg et al., 2012), and were therefore included as covariates in both the VBM and SBM analyses.

The AQS score was missing for one child. In line with recommendations for best practices for handling missing data, multiple imputation was employed to estimate the missing value (Enders, 2010) using the Markov Chain Monte Carlo procedure (Geyer, 1992) in SPSS software version 24.0 (IBM Corp., Armonk, NY, United States). Ten imputations were used and then averaged to maximize the precision of imputed data (Graham, 2009; Enders, 2010). To reach maximal accuracy, the imputations were performed based on the original 64 families using child sex and age at T1, as well as parental age and education at the time of recruitment as predictors in the imputation equation.

## Results

### Descriptive Statistics

At the time of initial recruitment (when children were 7 months old; *n* = 33), mothers and fathers were, respectively, on average 31.73 (*SD* = 5.10) and 33.27 (*SD* = 5.00) years old and had on average 15.36 (*SD* = 2.28; range 10–18) and 15.58 (*SD* = 2.00; range 11–17) years of education. The families’ average income fell in the $60,000 to $79,000 bracket. The majority of mothers (78.80%) and fathers (75.80%) were Caucasian. Most families had French as their first language (78.80%). Attachment security scores at T1 varied from -0.28 to 0.75 (*M* = 0.49, *SD* = 0.26).

At T2, almost half of the children (45%) were pre-pubertal, two were early pubertal (6%), 15 were mid-pubertal (45%), and one was post-pubertal (3%). Average brain volumetric data were: 1529.34 cm^3^ (*SD* = 106.58, range = 1317.63–1854.37) for total ICV; 833.27 cm^3^ (*SD* = 53.40, range = 725.37–987.76) for total GM volume; 442.40 cm^3^ (*SD* = 44.44, range = 367.07–552.58) for total WM volume; and 253.67 cm^3^ (*SD* = 23.68, range = 205.73–314.04) for total CSF. **Table 2** displays the bivariate correlations among attachment security, child age and sex, pubertal status, maternal education, and volumetric data (total ICV, total GM volume, total WM volume, and total CSF). No outliers were identified on any of the attachment or anatomical measures.

**Table 2:** Correlations between attachment security, average brain volumetric data, and covariates.

	1	2	3	4	5	6	7	8	9
(1) Attachment security									
(2) Total ICV	–0.09								
(3) Total GMV	0.05	0.94**							
(4) Total WMV	–0.30	0.92**	0.80**						
(5) Total CSF	0.04	0.66**	0.46**	0.48**					
(6) Child sex^a^	0.04	–0.53**	–0.53**	–0.51**	–0.25				
(7) Child age	–0.26	0.03	0.00	0.15	–0.12	–0.23			
(8) Maternal education	0.21	–0.31	–0.27	–0.25	–0.31	0.21	–0.10		
(9) Pubertal status^b^	0.13	–0.38*	–0.39*	–0.33t	–0.21	–0.16	0.69***	0.11	

*^a^Child sex was coded 1 = boy; 2 = girl. ^b^Pubertal status was coded 1 = prepubertal; 2 = early pubertal; 3 = mid pubertal; 4 = late pubertal. ICV, intracranial volume; GMV, gray matter volume; WMV, white matter volume; CSF, cerebrospinal fluid. ^t^*p* < 0.10, ^∗^*p* < 0.05, ^∗∗^*p* < 0.01, ^∗∗∗^*p* < 0.001.*

### Voxel-Based Morphometry

**FIGURE 1:**
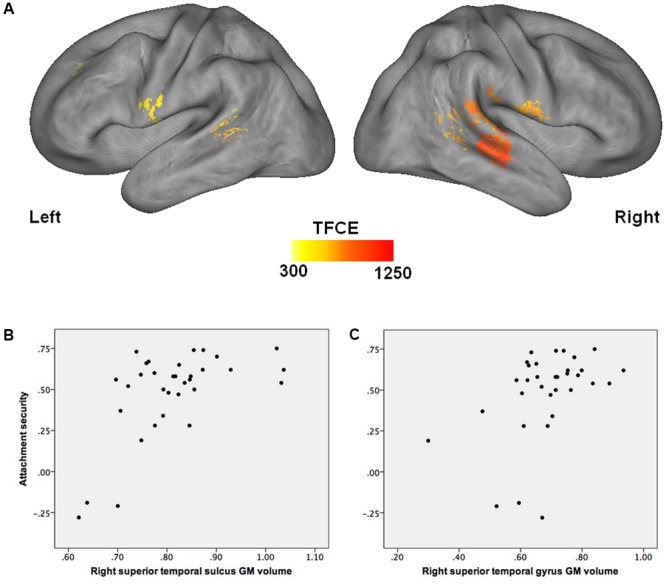
Association between attachment security in infancy and GM volume in late childhood. **(A)** Higher attachment security in infancy is associated with greater GM volume in the right superior temporal sulcus and gyrus, temporo-parietal junction, and precentral gyrus, as well as in the left superior temporal sulcus and precentral gyrus (FDR corrected, *p* < 0.05), after accounting for child age, sex, pubertal status, maternal education, and total intracranial volume. **(B)** Correlation between attachment security in infancy and GM volume in the right superior temporal sulcus in late childhood [*x* = 45; *y* = –21; *z* = –4]. **(C)** Correlation between attachment security in infancy and GM volume in the right superior temporal gyrus in late childhood [*x* = 68; *y* = –33; *z* = 20]. GM, gray matter.

Multiple regression analysis indicated that after accounting for child age, sex, pubertal status, maternal education, and total ICV, children who were more securely attached to their mother in infancy had larger GM volumes in the right hemisphere covering the superior temporal sulcus and gyrus, extending to the middle temporal gyrus, and into the temporo-parietal junction. Increased GM volume in the left superior temporal sulcus and in the bilateral precentral gyri was also related to higher attachment quality (see **Figure 1** and **Table 3**). No significant supra-threshold voxels were found for negative contrasts.

**Table 3:** Regional volumes significantly associated with attachment security in infancy (*p* < 0.05, False Discovery Rate correction).

Regions	BA	*k*	MNI coordinates (*x, y, z*)	*TFCE*
**Right**				
Superior temporal sulcus^a^	48/21	601	45, -21, -4	1122.72^∗^
			57, -24, -3	1120.15^∗^
Superior temporal gyrus	48	150	68, -33, 20	816.95
Temporo-parietal junction	21	34	60, -39, 2	744.62
			60, -48, 9	735.12
Precentral gyrus	48	43	63, -3, 9	649.53
**Left**				
Superior temporal sulcus	22	22	–58, -40, 8	660.78
Precentral gyrus	48	51	–52, -3, 15	450.97

*^∗^Results hold at *p* < 0.05, Family Wise Error correction. ^a^Cluster peak was in the superior temporal sulcus, but the cluster also covered the superior and middle temporal gyrus. BA, Brodmann area; *k*, number of voxels; *TFCE*, threshold free cluster enhancement statistic.*

### Surface-Based Morphometry

Multiple regression analysis indicated that attachment security in infancy was not significantly related to cortical thickness in late childhood, neither positively nor negatively, over and above child age, sex, pubertal status, and maternal education (*p* > 0.001, uncorrected).

## Discussion

To our knowledge, this is the first study to examine the prospective associations between attachment security in infancy and whole-brain GM volume and thickness in childhood. The main findings indicate that children who were more securely attached to their mother in infancy (15 months) had larger GM volume in the bilateral superior temporal sulci, right superior temporal gyrus, right temporo-parietal junction, and the bilateral precentral gyri in late childhood (10–11 years). These results survived a multiple comparisons correction after controlling for several potentially confounding variables. No significant association was found between attachment security and cortical thickness. Consistent with animal studies indicating that enriched caregiving is associated with optimal brain development (Greenough et al., 1987; Van Praag et al., 2000; Meaney, 2001), the current study provides rare data in humans consistent with the idea that attachment relationships may affect children’s brain development, as reflected by larger GM volume in the frontal and temporal lobes. Moreover, these results contribute to the emerging literature indicating that variations in the quality of caregiving experiences within the normative range are associated with child brain morphology (Luby et al., 2012, 2016; Whittle et al., 2014, 2016; Kok et al., 2015, 2017).

Specifically, better quality mother–child relationships in infancy were found to be predictive of larger GM volume in the superior and middle temporal gyri, superior temporal sulci, temporo-parietal junction, and precentral gyri in late childhood. This appears to be the first evidence of a relation between a direct observational measure of caregiving quality (whether parenting or attachment) and GM volume in these specific brain regions. The lack of prior comparable findings may be partly expected, given that no previous studies have investigated the association between parent–child relationship quality or parental behaviors and *whole-brain* GM volume in a pediatric community sample. However, the current findings are broadly consistent with studies reporting smaller GM volume or surface in the superior and middle temporal gyri of maltreated children (Hanson et al., 2010; De Brito et al., 2013; Kelly et al., 2013, 2015; Lim et al., 2014). These areas are critical for processing emotional stimuli (Allison et al., 2000), a function that is impaired in maltreated children, as indicated by event-related potential and functional MRI studies (see da Silva Ferreira et al., 2014 for a systematic review; Pollak et al., 2001). Importantly, the preliminary results presented here suggest that even normative variations in relationship quality may have a long-lasting impact on the development of these brain regions. This is a promising first step, but independent replication is necessary.

In contrast to previous studies reporting an association between parental behavior and cortical thickness in typically developing children (Whittle et al., 2014, 2016; Kok et al., 2015), no significant association was found in this sample between attachment security in infancy and cortical thickness in late childhood. Methodological differences, such as the modest sample size and related limited statistical power in the current study, may account for this discrepancy. Developmental considerations may also be at play. Brain volume and thickness follow an inverted U-shape developmental trajectory characterized by an increase during childhood, a region-specific peak in late childhood and early adolescence, and a subsequent decrease (Shaw et al., 2008; Giedd et al., 2015). Results reported here are age-specific (10–11 years); in a younger or older sample, results may be different. Previous work by Kok et al. (2015) indicates that maternal sensitivity in early childhood is associated with brain volume and thickness in 8-year-old children. Moreover, as Teicher and Samson (2016) underscore, caregiving experiences may not relate to brain structures at one specific period of development, but rather, they may be associated with the trajectory of brain development over time (see also Whittle et al., 2014). Thus, one intriguing possibility that could be investigated in future work is that attachment security may not relate to cortical thickness at specific ages, but rather to the rhythm of cortical thickening and then thinning over time. Alternatively, the findings may be theoretically meaningful, indicating for instance that, although related to an extent, parenting behavior and parent–child attachment may have a different impact on brain development. Of note, we did not find links between attachment and amygdalar volume as observed by Moutsiana et al. (2015) and Lyons-Ruth et al. (2016). In addition to the different composition of the samples studied and the different attachment measure used, developmental considerations may again underlie discrepant findings, given that the previous studies found links between early attachment and amygdalar volume in adulthood. Longitudinal designs including repeated MRI would be useful to more accurately depict the developmental aspects of the brain-attachment associations.

### Attachment and the Developing Brain: Proposed Mechanisms

Children more securely attached to their primary caregivers are exposed to a variety of experiences that differ from those characterizing insecurely attached children. These experiences may influence children’s brain development in regions involved in social, cognitive, and emotional functioning. A central way in which the experience of securely attached infants differs from that of their insecurely attached counterparts is with regards to the quality of the emotion regulation provided by the caregiver. Indeed, one of the hallmarks of a secure attachment relationship is the caregiver’s capacity to provide adequate external regulation when the infant encounters an affectively challenging situation during exploration (e.g., frustration when faced with a complex toy, fear of a large dog in the park). As a result, securely attached children are exposed to repeated experiences of successful regulation in emotionally taxing situations, which provides a strong basis for the gradual development of self-regulation (Calkins, 2004; Cole et al., 2004). The superior and middle temporal gyri are activated when subjects need to down-regulate their negative affect (Ochsner et al., 2004; Frank et al., 2014), and extensive evidence from human and non-human primates points to a crucial role for the superior temporal gyrus and sulcus for processing emotional faces stimuli (Britton et al., 2006; Pagliaccio et al., 2013). If replicated, the current findings would suggest that the repeated experiences of successful emotion regulation that characterize secure attachment relationships may promote optimal development in brain regions that subsume socio-emotional regulation, such as the superior and middle temporal gyri, through experience-dependent processes.

An alternative hypothesis for the observed relation between attachment and brain structure pertains to one of the central notions of attachment theory, that of “internal working models” (Bowlby, 1969/1982, 1973, 1980, 1988). These models consist of mental representations of self and others, which are thought to be shaped by daily interactions with primary caregivers. The repeated experiences of responsive care that characterize secure attachment are believed to promote the development of positive internal working models of self and others (Bretherton and Munholland, 2016). It is theorized that these models are progressively internalized, becoming an integral part of the child’s personality, and are increasingly generalized to new relationships, guiding behavior and interpretation in new social situations and helping children correctly anticipate future social interactions (Bretherton and Munholland, 2016). Empirical evidence indeed shows that securely attached infants develop positive expectations about social interactions (Johnson et al., 2010; Biro et al., 2015). Importantly, the superior and middle temporal gyri, temporo-parietal junction, and precentral gyrus are involved in the representation and elaboration of past and future events (Kelley et al., 2002; Addis et al., 2007; Spreng et al., 2009; Holland et al., 2011; Jacques et al., 2011) and representation of self and others (Ruby and Decety, 2001; Ochsner et al., 2004). The positive expectations about social relationships characterizing secure attachment working models may lead securely attached children to engage more confidently in social interactions. Thus, these children are likely to be more frequently engaged in stimulating social interactions which may result in recurrent activation of brain regions involved in the representation of self and others in social contexts. As such, secure attachment could promote the optimal structural development of the superior and middle temporal gyri, temporo-parietal junction, and precentral gyrus.

Lastly, social perception and social cognition may play a role in the attachment-brain structure links uncovered here. Social perception is an important basis for the development of attachment relationships. In order to effectively attain a caregiver’s proximity, children have to adapt their attachment behaviors toward their caregivers according to the context, caregiver location, and the specific characteristics of the caregiver with whom they are interacting (Cassidy, 2016; Sroufe, 2016). Recognizing the caregiver’s face and affective state as well as following his or her eye gaze and movements support the contextual adaptation of infant attachment behavior for proximity seeking. As such, empirical evidence indicates that higher levels of attachment security in infancy are associated with better emotion recognition skills up to 10 years later (Steele et al., 2008). Assuming that future research replicates the current results, the association between attachment security and GM volume in the superior and middle temporal gyri may therefore be related to the importance of these brain regions for social perception, such as the detection of faces, eye gaze, and biological motion (Puce et al., 1998; Allison et al., 2000; Haxby et al., 2000; Engell and Haxby, 2007; Saygin, 2007). It is possible that securely attached children are more successful in adapting their attachment behaviors to the context by recruiting temporal regions involved in social perception, which in turn promotes the development of these regions. The value of attachment security for complex social cognitive processes and social functioning is also well established (Thompson, 2016), and numerous studies have underscored the role of the superior temporal sulcus and gyrus, middle temporal gyrus, and the temporo-parietal junction in theory of mind, moral reasoning, and empathy (see Bzdok et al., 2012, for a meta-analysis). Larger GM volumes in the superior and middle temporal gyri have been related to more optimal social skills, such as better emotion recognition (Shdo et al., 2017) and better ability to predict others’ behavior based on mental states (Powell et al., 2014). Conversely, reduced GM thickness or volume in the superior and middle temporal gyri has been associated with lack of empathy and compassion and severity of conduct disorder symptomatology (Huebner et al., 2008; Fahim et al., 2011; Wallace et al., 2014). Overall, these studies suggest that GM volume and functional activity in the superior temporal sulcus and gyrus, middle temporal gyrus, and temporo-parietal junction are closely linked with social cognition and social functioning, of which attachment security is a well-known predictor (Thompson, 2016). Social cognitive experiences embedded in secure attachment relationships provide children with a more sophisticated understanding of the psychological dimensions of social interactions (Thompson, 2016) and may therefore contribute to shaping the structural development of regions involved in social cognition, such as the superior temporal sulcus and gyrus, the middle temporal gyrus, and the temporo-parietal junction.

### Limitations

The results presented here must be interpreted in the context of some limitations. First, the longitudinal but non-experimental design precludes causal inference and determination of directionality. The possibility that larger GM volumes in the superior and middle temporal gyri, the superior temporal sulci, the temporo-parietal junction, and the precentral gyri were already present in these children in infancy, and may have predisposed them to develop secure attachment to their mothers, cannot be excluded. In fact, given that developmental processes are transactional by nature (Sameroff, 2009), it is reasonable to expect that any caregiving-brain associations are probably bidirectional, reflecting the action of mutual reciprocal influences between parent and child (Serbin et al., 2015). The non-experimental design also leaves open some third-variable explanations, notably the possibility that shared genes between mother and child may be partly responsible for the links observed. This is unlikely to have played a major role in the current results though, given that several genetically informed studies show that the variance in mother–child attachment security (O’Connor and Croft, 2001; Bokhorst et al., 2003; Roisman and Fraley, 2008) and the variance in maternal caregiving behavior (Roisman and Fraley, 2008) are almost entirely attributable to environmental influences, with small to negligible genetic contributions. Other third-variable explanations are possible though, one of which being that caregiving experiences (e.g., exposure to higher parental sensitivity) could influence both the quality of attachment relationships and the development of corresponding brain regions. Second, the small sample size and the use of several covariates reduced statistical power, potentially leading to underestimation of the links between attachment and brain volumes and thickness. Clearly, replication in larger independent samples is necessary to confirm the links reported here, especially for clusters in the right temporo-parietal junction and precentral gyrus, as well as in the left precentral gyrus and superior temporal sulcus, due to the small number of voxels contained in these clusters. Third, the attachment measure used in the current study does not allow the assessment of attachment disorganization (the most extreme form of attachment insecurity, assessed exclusively through the SSP), which one study found to be related to amygdalar volume (Lyons-Ruth et al., 2016). Disorganized attachment relationships are associated with the development of psychopathology, poor emotion regulation skills, and poor relationships with peers and adults (Lyons-Ruth and Jacobvitz, 2016), which could be reflected in children’s brain morphology, especially in brain regions known to be involved in socio-emotional functioning (limbic system, social brain). Fourth, we did not assess father–child attachment security, which may differentially influence brain development given that fathers have unique contributions to children’s social and cognitive development (see Cabrera and Tamis-LeMonda, 2013). However, Kok et al. (2015) reported that the associations between parental sensitivity and brain morphology were similar for mothers and fathers in their sample.

## Conclusion

This 9-year longitudinal study suggests that better mother–child attachment quality in infancy is related to greater GM volume in the superior temporal sulcus and gyrus, the temporo-parietal junction, and precentral gyrus in late childhood, whereas no associations with measures of cortical thickness were found. This appears to be the first study to investigate the link between infant-caregiver attachment quality and brain morphometry in childhood. The use of a gold-standard observational measure of attachment security in the ecological context of the family home, along with whole-brain analyses using a pediatric template, enabled the identification of novel associations between attachment and brain regions involved in social, cognitive, and emotional functioning, and these associations were robust to several important covariates. Although preliminary and in need of replication, the present results provide further evidence that infant quality of attachment toward a primary caregiver is important not only for children’s social, emotional, and cognitive functioning, but may also be involved in their brain development. As in previous studies focusing on maltreatment (e.g., da Silva Ferreira et al., 2014; Puetz et al., 2017), future research in normative samples could test, using other methodologies (e.g., diffusion tensor imaging, functional connectivity, event-related potentials), the breadth of the links between attachment security in infancy and brain morphology and functions.

## Ethics Statement

The research ethics committee of aging-neuroimaging of the CIUSSS du Centre-Sud-de-l’île-de-Montréal approved the study. The goal of the study was explained to children and their parents, who signed a detailed inform consent form. We report how we determined our sample size, all manipulations, and all measures in the study.

## Author Contributions

ÉL participated in data collection, performed statistical analyses, and drafted the initial manuscript. AB designed the study, wrote parts of the manuscript and revised it for intellectual content. MB contributed to designing the study, edited and revised the manuscript for intellectual content. FD contributed to data analyses and interpretation, wrote parts of the manuscript and revised it for intellectual content. VD participated in data collection and methodological choices, and revised the manuscript for intellectual content. All authors gave their final approval of the manuscript to be published and agreed to be accountable for all aspects of the work.

## Conflict of Interest Statement

The authors declare that the research was conducted in the absence of any commercial or financial relationships that could be construed as a potential conflict of interest.

## Footnotes

1 www.neuro.uni-jena.de/cat/

2 www.bic.mni.mcgill.ca/ServicesAtlases/NIHPD-obj1
